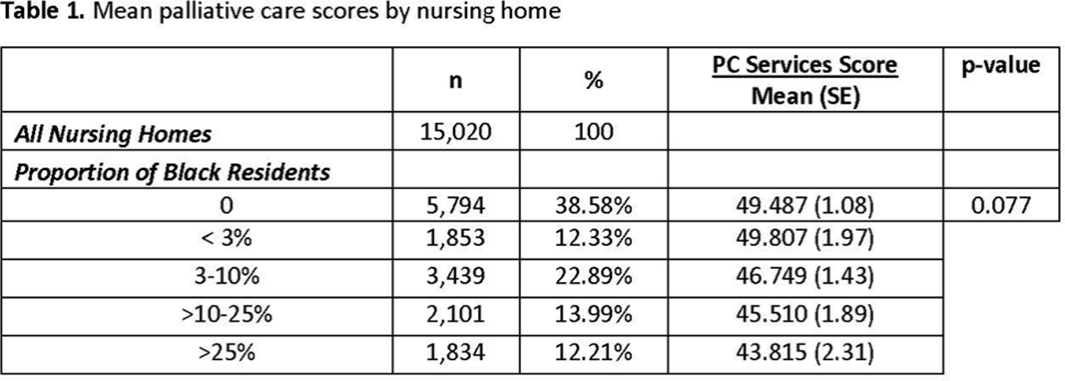# Racial Differences in Infection Management and Palliative Care at End-of-Life in Nursing Homes Nationwide

**DOI:** 10.1017/ash.2021.133

**Published:** 2021-07-29

**Authors:** Leah Estrada, Andrew Dick, Patricia Stone, Jordan Harrison

## Abstract

**Background:** Infections are common at end-of-life in older nursing-home residents. This often leads to the overuse of antibiotics and burdensome treatments. Improving infection management through palliative care at the end of life has been proposed as a key strategy to reducing inappropriate antibiotic use. Black nursing-home residents tend to reside in poorly performing nursing homes. We examined palliative care services in nursing homes with varying proportions of black residents. **Methods:** Cross-sectional, nationally representative nursing-home survey data (2017–2018) was combined with the Minimum Data Set 3.0 (nursing-home resident characteristics), the Certification and Survey Provider Enhanced Reporting data (nursing-home facility characteristics), and the Multidimensional Deprivation Index (county-level poverty estimates). The survey included 24 validated items on nursing-home palliative care services, as well as the nursing home’s infection control program and integration of infection management and palliative care (summative score, 0–100). We used nursing-home facility-level multivariate regression to estimate the relationship between proportion of black residents and palliative care scores, before and after controlling for county-level poverty estimates, facility characteristics, and resident characteristics. We categorized proportion of black residents using methods reported in the literature (25%). **Results:** The mean weighted palliative-care score in our sample of 869 nursing homes (weighted n = 15,020) was 47.7 (SE, 0.70). In unadjusted analyses, nursing homes with higher proportions of black residents provided significantly fewer palliative care services than nursing homes with no black residents, with the greatest differences (*P* = .027) observed between nursing homes with >25% black residents (mean palliative care score, 43.82; SE, 2.31) versus nursing homes with no black residents (mean palliative care score, 49.47; SE, 1.08). These disparities persisted after adjustment for urbanicity and county-level poverty rates (p < 0.01) but were attenuated after further adjustment for resident and facility level characteristics (p=0.138). **Conclusions:** Our findings demonstrate that wide variations in nursing-home palliative-care services exist with increased proportions of black residents, even after accounting for community characteristics. Further research is needed to identify and understand the specific community characteristics that play a role in the provision of palliative care services. Palliative care is a method to reduce inappropriate antimicrobial use at the end of life and should be expanded with a focus on nursing homes with higher proportions of black residents.

**Funding:** No

**Disclosures:** None

**Table 1.**